# Diversifying academic medicine: One search committee at a time

**DOI:** 10.3389/fpubh.2022.854450

**Published:** 2022-08-19

**Authors:** N. Nicole Jacobs, Jovonnie Esquierdo-Leal, Gregory S. Smith, Melissa Piasecki, Ramona A. Houmanfar

**Affiliations:** ^1^University of Nevada, Reno School of Medicine, Reno, NV, United States; ^2^Department of Psychology, University of Nevada, Reno, NV, United States

**Keywords:** diversity and inclusion, healthcare workforce diversity, racial and gender diversity, implicit bias training, faculty diversity, search committee training

## Abstract

Despite increasing attention to lack of diversity among medical education faculty, those traditionally underrepresented in medicine remain so. In 2017, the University of Nevada, Reno School of Medicine approved a new policy to increase diversity in the faculty search process, which includes a mandatory 2-h workshop on best practices in search processes and implicit bias training. Workshop participants were 179 search committee members making up 55 committees from February 2017 to March 2020. Participants completed two separate social validity surveys, one immediately following the workshop and another following the close of their search, and rated various aspects of the workshop. Each search committee completed a Diversity Checklist (DCL) of various mandatory and best practices to be implemented during each search. Historical data on diversity of job applicants, interviewees, and hires over the 5-year period immediately preceding workshop implementation were compared with corresponding diversity data from the participant search committees for a 3-year period following implementation of the workshop. Social validity surveys indicated high ratings pertaining to the benefits of the workshop (means 3.82–4.39 out of 5). Implementation of practices outlined in the DCL were high (94% of mandatory and 87% of best practices). Chi-square analyses of diversity data before and after implementation revealed significant increases in overall diversity (both race and gender) of applicants (*p* < 0.001), interviewees (*p* = 0.002), and those offered a position (*p* = 0.002), in the time period following implementation. Follow-up comparisons found greater increases for gender relative to race/ethnicity.

## Introduction

The AAMC (1, 2) advocates for increasing diversity in academic medicine, highlighting the relationship between diversity and excellence in medical education (3–8), improved access to care and better patient care outcomes (9–15), advances in research (16–18), and better management decisions with a more diverse workforce (19, 20). Scholars in public health have also highlighted the lack of diversity in the field and the need to diversify the workforce (21, 22). Despite these compelling arguments and calls to increase diversity in academic medicine, faculty who are Underrepresented in Medicine (URM) constitute only 7.3% of all faculty in US medical schools and women constitute only 41.6% (23, 24). Faculty search committees operate as gatekeepers to academic medicine and a rich literature attests to how racial and gender bias may participate in search committee decision making (25–27).

In order to address the potential negative effects of bias on search committees, the AAMC (28) and others (29) recommend several strategies, including implicit bias training. Recommended best practices to address the potential of implicit bias include individual search committee members completing assessments of their implicit bias and engaging in implicit bias training. Although implicit bias training for search committees is a gold standard, it has been criticized for its lack of effectiveness over time (30) and is not a sufficient tool to create lasting change in search committee members. Accordingly, implicit bias training to address interpersonal bias should be complemented with additional strategies that address bias at the systemic level. Best practices to address the potential of systemic racism consist of ensuring a diverse composition of the search committee, clearly defining required and preferred criteria for candidate selection, deepening the pool of candidates through active recruitment strategies, utilization of de-identified grids to equitably compare candidates based on required and preferred criteria, use of candidate diversity statements, implementation of objective and structured behavioral interviewing questions to interview candidates, evaluation of candidates using objective rubrics, providing high-level customer service to ensure an inclusive experience during candidate visits, and inclusive onboarding of selected candidates (28).

Given the aforementioned strategies are reported as “best” and “proven” practices (28), academic institutions have invested significant time and resources into their implementation. However, there is a paucity of literature pertaining to a number of key metrics associated with these recommendations, including feasibility of implementation, acceptance by faculty members, faculty compliance with recommendations following training, and most importantly, the outcome of increasing the diversity of candidates sourced, interviewed, and hired (31). Despite greater societal attention to diversity and inclusion and efforts within medical education to increase diversity, the AAMC Faculty Roster (23, 24) shows little improvement in diversity in academic medicine. A systemic approach to increase diversity and equity at all stages of the search process, incorporating best practices touted in the literature with an ability to assess and improve outcomes, is clearly needed to increase the diversity of the workforce in academic medicine.

In addition, intentional focus on communication of organizational changes (i.e., policy and/or process) and complementary training are critical. Research suggests that diversity and implicit bias training may contribute to diversity resistance among those who identify with non-marginalized groups (32). Diversity resistance refers to the interrelation between individual resistance, rooted in implicit bias and motivation, and organizational practices that perpetuate bias and exclusion (32). Endorsing values that oppose prejudice may reduce resistance as it motivates one to seek information and engage in behaviors that support those values (32, 33). Therefore, making structural and organizational changes by targeting process, while also pinpointing attendee acceptance is key to reducing barriers contributing to bias in academic searches.

In February 2017, The Faculty Search Policy to Create Excellence and Diversity was passed and implemented at the University of Nevada, Reno School of Medicine (UNR Med). The policy addresses both systemic and interpersonal racism by mandating that all members of search committees complete a 2-h, in-person workshop on best practices, including training on implicit bias, to increase diversity equity. The workshop incorporates the best practices noted above, as described in the literature. Values alignment, imagery, and goal setting are also incorporated to address motivation and promote values-based behaviors (33, 34). It is important to note that none of these best practices were systematically implemented prior to the implementation of the new policy.

The purpose of this study was to evaluate the participatory influence of the search committee diversity training package on committee members' perceived utility and on the diversity of applicants hired at UNR Med for all faculty positions, including leadership positions. We predicted that the workshop would be well-received by participants and feasible for an institution to adopt as a mandatory component of the search committee process. We also anticipated that participants would report high levels of intent to implement the practices upon completion of the workshop and high levels of actual implementation of the practices upon completion of their respective searches. Finally, we hypothesized that in the time following implementation of the workshop, the diversity (race and gender) of the pool of candidates at each stage of the search process would be greater than during the years prior to implementation of the workshop.

## Method

The 2-h, in-person search committee workshop was provided separately for each search committee, at the outset of that respective search. The workshop included content on (1) How diversity fits into the vision, mission, and core values at UNR Med; (2) The relationship between diversity and excellence, including a review of data on how diversity enhances medical education, influences access and outcomes in health care, advances research, and improves management decisions (6, 9, 18, 20); (3) Implicit Bias Training, including definitions and examples of implicit bias and how it differs from explicit bias, experiential exercises to elicit and discuss implicit biases, a discussion of participants' implicit bias test results [utilizing the Implicit Relational Assessment Procedure (IRAP)] (35) and how to interpret their data, a review of the literature on how implicit bias has been shown to affect decision making in search committees as well as promotion and tenure committees in academia, and a review of best practices to be aware of and manage bias (36), such as mindfulness (37–39), perspective-taking (40, 41), and focusing on shared values and goals (42); and (4) Methods to improve diversity at each stage of the search process, as presented in the Diversity Checklist (DCL, see Table 1), including creating a diverse search committee, guidance on inclusive language in advertising, tips to diversify the applicant pool, equitable review of applicants, use of standardized interview questions, inclusive on-campus visits, and equitable selection of final candidates through use of rubrics. Handouts relating to these best practices, such as sample questions to assess candidates' background in diversity and inclusion and the DCL, were provided in a booklet to all participants. If search committee members were not able to attend the training or make-up trainings, they were removed from the search committee, as per our policy. The study was approved through the university's Institutional Review Board.

**Table 1:** Diversity checklist guidelines established to identify best practices and promote diversity in the search process.

**Pre-search**
Minimum and preferred qualifications are clearly defined.^*^
**Search committee selection**
Identify a diverse committee, with at least two members from our school-identified diversity categories.^*^
**Preparing search committee members**
*Hiring authority reviews job description, including minimum and preferred qualifications*.
**Running an effective/efficient search committee**
*Establish ground rules around conversations on diversity*.
*Discuss the importance of diversity at each stage of the hiring process*.
Each search committee member attends training on diversity and implicit bias.^*^
**Advertising**
Language in advertisements includes diversity statement and proactive statement to convey institutional commitment to diversity.^*^
*Language is gender neutral*.
**Tips for a diverse pool**
Advertisements are placed on diversity websites (a minimum of two).^*^
Proactive outreach to potential candidates from school-identified diversity categories (cold calls, emails, networking, pipelines, partnerships, professional meetings and
organizations, etc.). Ask Office of Professional Recruitment for assistance with sourcing, if necessary.^*^
**Reviewing applications**
Use a rubric (templates available) to evaluate each candidate. Each rubric includes a section for record on diversity.†
Review each candidate's diversity statement and add diversity experience/competence to evaluation grid.†
*Create a grid of candidates based on established criteria, minimal and preferred qualifications*.
*Use blind/de-identified grids*.
*Consider the entire application (whole candidate)*
**Interview questions**
Questions map onto position qualifications.†
Ask questions to assess candidate's experience with and competencies in diversity. Avoid unacceptable inquiries.†
Use a rubric to evaluate each candidate on each question.†
**On-campus visits**
*Ask the candidate if there are people they want to meet with and places they want to see, and arrange meetings/tours if possible*.
*Consider a tour of UNR*'*s Diversity Center*.
*Consider a tour of Reno, including cultural and faith communities as desired by the candidate*.
*Refer candidate to community resources as requested by them*.
**Evaluating/ selecting final candidates**
*Use rubrics that map onto job qualifications/competencies. Rubrics should include evaluation of diversity record/competency*.
*Get quantitative and qualitative data on candidates from interviewers*.
Search committee members are careful to avoid bias in evaluating and selecting candidates.†
The search committee recommends an onboarding peer once a final candidate is selected.†
The hiring authority engages the onboarding peer.†

Stages of search process in bold headings.

* Mandatory practices, †Best Practices, Suggested practices (italicized).

Search committee member participants included classified staff, faculty, and community partners. Two different social validity surveys (SV1 and SV2, see Table 2) asked participants to rate several aspects of the workshop and its utility on a 5-point scale. Social validity measures seek participants' perspectives in determining if behavior change is socially meaningful and consider their vantage points in measuring the benefits and costs of behavior change efforts (43). As is common with social validity measures, we developed these measures in house (as opposed to using a pre-existing measure) and tailored them to obtain data specific to the core aspects of the new training and related search committee processes.

**Table 2:** Means and standard deviations for post-workshop social validity surveys (SV1 & SV2).

**Social Validity 1 (*n* = 112)**	**Mean (SD)**
**Insight**	4.4 (0.7)
I understand the concept of implicit attitudes and how it relates to my participation in the search process.	4.6 (0.5)
The workshop made me more aware of my biases	4.1 (0.8)
I understand the relationship between diversity and excellence in hiring new faculty.	4.5 (0.6)
The workshop on implicit bias provided insight on my implicit bias and how to manage it in the search process.	4.2 (0.7)
**Action**	3.9 (0.9)
The results of my IRAP will influence my actions as a search committee member.	3.5 (0.8)
The workshop has motivated me to learn more about implicit bias	4.0 (0.9)
I believe the workshop will influence my actions as a search committee member.	4.2 (0.7)
**Delivery**	4.4 (0.7)
The objectives of the workshop on implicit bias were clear	4.4 (0.6)
The facilitator was knowledgeable and was able to teach the material effectively.	4.6 (0.5)
The time allotted to the workshop on implicit bias (including assessments) was reasonable.	4.1 (0.8)
I would recommend the training to other faculty.	4.4 (0.7)
**Usefulness**	4.2 (0.7)
The suggestions for how to promote diversity at each step of the search process will be useful.	4.2 (0.7)
Materials provided by the trainer (e.g., sample interview questions, sample evaluation templates, sample candidate evaluation	4.2 (0.8)
spreadsheet, etc.) will be useful in my work.
Checklists for promoting diversity at each stage, provided by the trainer will be useful in my work.	4.3 (0.7)
Overall, I found the training to be useful.	4.3 (0.7)
**Social Validity 2 (*****n*** **=** **51)**
**Insight**	3.9 (0.9)
The training made me more aware of my biases	4.0 (0.9)
The training motivated me to learn more about implicit bias	4.0 (0.9)
Overall, I believe completing the IRAP made me more aware of my biases throughout the search process	3.8 (0.9)
Completing the IRAP made me more conscious of my biases entering the faculty interviews	3.7 (0.9)
**Action**	3.8 (0.9)
The Workshop on Implicit Bias influenced my actions as a search committee member	3.7 (0.9)
I pay more attention to implicit biases in my decision-making at work	4.0 (0.9)
I found that the Workshop on Implicit Bias made it easier for me to consider biases as I participated in the search process	3.8 (0.9)
I used tips provided in the Workshop on Implicit Bias throughout my participation in the search	3.9 (0.9)
**Checklist**	4.2 (1.0)
I found that the checklist was helpful in ensuring diversity topics were considered at each stage in the search process	4.3 (1.0)
I found the checklist useful to the search process	4.0 (1.1)
I would recommend the use of the checklist to other search committees.	4.2 (1.0)
Materials provided by the trainer (e.g., sample interview questions, sample evaluation templates, etc.) were useful to the search committee.	4.5 (0.9)

Row headers (e.g., Insight) indicate groupings of certain items based on content, with individual items listed below each.

Immediately following the workshop, participants were emailed a link to an online form of SV1, which included questions on their satisfaction with the workshop, how useful they found the content and handouts, development of insight, and intent to implement strategies and tools provided in the workshop. Throughout each search, the Office of Professional Recruitment (OPR) communicated with each respective search coordinator, in order to ensure compliance with the mandatory items in the Diversity Checklist (see Table 1). Thus, the DCL provided longitudinal instructions plus expectations for search committees to follow the best practice, and increase diversity throughout the search process. If search committees did not complete mandatory items on the DCL, the search was halted by OPR until compliance was met, as per our Faculty Search Policy to Create Excellence and Diversity, which provided a longitudinal contingency to promote adherence to best practices. Once the position was filled and the search was complete, a link to SV2 was sent to search committee members, which allowed them an opportunity to report in retrospect on the usefulness and actual implementation of the strategies and tools offered in the workshop, as well as their evolving satisfaction with the workshop. Although not every search was identical in duration, SV2 was administered to participants immediately following the close of their respective search.

Moreover, historical data on the diversity of candidates for the 5-year period (1 January 2012 through 31 January 2017) prior to implementation of the new workshop were gathered and compared to similar data for the immediate 3-year period (February 2017 to March 2020) following implementation of the workshop, in order to determine if the new workshop corresponded with an increase in the diversity of applicants, candidates interviewed, and hires.

## Results

A total of 179 participants completed the workshop and were therefore asked to complete the first social validity survey following the workshop. The vast majority of these participants ultimately participated in at least one search, however, some participants were part of multiple searches, while others may have left the institution before or during a respective search, given that searches were typically 6 months in duration. It is important to note that every search committee was a unique combination of members, although a specific committee member may have participated in more than one unique search committee over the entire 8-year span of the study period. If a search committee member participated in more than one search, they were only required to complete the workshop once, as the Faculty Search Policy to Create Excellence and Diversity mandates renewal of the training every 3 years. The 179 participants who received the training package constituted 89% (179/201) of individuals who were mandated to attend, suggesting a positive response to mandatory training and indicating the feasibility of making such training mandatory for all search committee members. As noted previously, if a search member did not complete the mandatory training, they were removed from the search committee.

The first social validity survey (SV1, see Table 2) was completed by 112 of 179 participants (62% response rate), who indicated the workshop was generally well received. The subset of questions pertaining to satisfaction with delivery of the workshop were rated highly, mean (SD) = 4.4 (0.7) on a 5-point scale. Other subsets of questions were rated similarly: level of insight gained, mean (SD) = 4.4 (0.7); intent to take action immediately after the workshop, mean (SD) = 3.9 (0.9); and usefulness of the workshop, mean (SD) = 4.2 (0.7). The second social validity survey (SV2, Table 2) was sent to 136 participants, 51 of whom responded (37% response rate). Data from SV2 remained positive, with high ratings on questions pertaining to insight gained by completing the IRAP implicit bias assessment, mean (SD) = 3.9 (0.9); applying the techniques and tools presented in the workshop, mean (SD) = 3.8 (0.9), and evaluations of the DCL, mean (SD) = 4.2 (1.0).

Data from the DCL indicated high levels of implementation of the practices taught in the workshop, based on yes/no responses of whether specific practices listed in the DCL were implemented during each respective search. Only one DCL was required per search committee and was submitted by the search coordinator. Of the total search committees that participated, 91% (50/55) completed and submitted the DCL. Compliance with both mandatory practices (94% average) and best practices (88% average) listed in the DCL was high.

**Figure 1:**
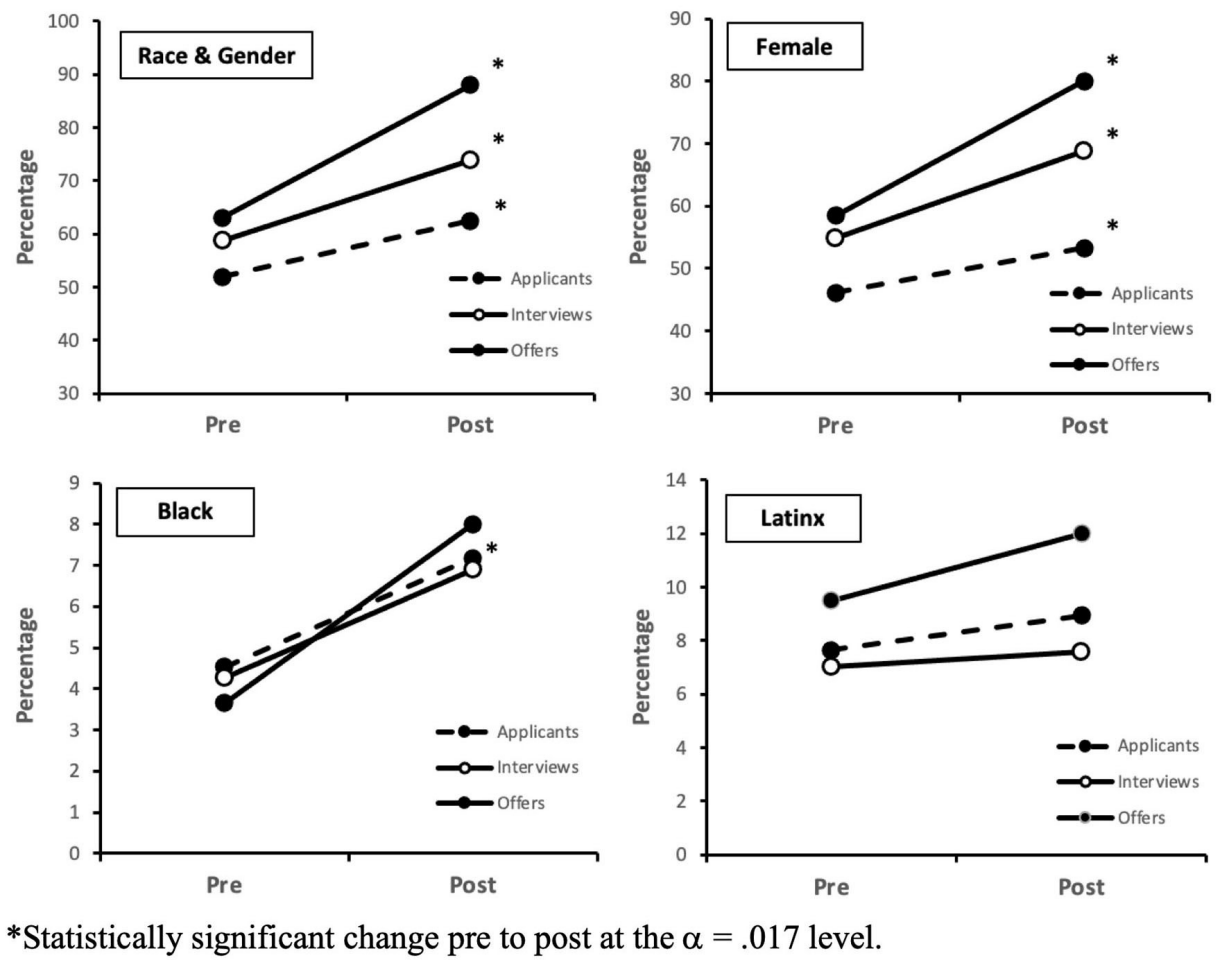
Percentage of diverse individuals by category among applicants, interviewees, and offers. *Statistically significant change pre to post at the α =0.017 level.

The most important outcome variable was whether the diversity of the pools of candidates improved once we began implementing the workshop, which was evaluated through a pre-post design. Percentages of diverse candidates pre- and post-workshop throughout the search process are depicted by category in Figure 1. Chi-square analyses were used to determine whether the proportions of diverse individuals at each stage of the search process were different prior to and following implementation of the workshop. As hypothesized, findings indicated increases in the diversity (race and gender) of candidates throughout the search process, beginning with applicants to each position, then applicants selected for interviews, and eventual hires (Table 3). Follow-up comparisons (employing Bonferroni corrections for alpha) were utilized to compare demographic diversity data before and after the workshop based on several diversity categories: gender (female) only, combined race (Black and/or Latinx), and Black and Latinx separately.

**Table 3:** Chi-square analyses of diversity of faculty job applicants, applicants offered interviews, and those offered the job, before and after implementation of faculty search committee workshop.

	**Pre**		**Post**				
	***N***	**%**	***N***	**%**	**χ^2^**	**df**	***P***
**Applicants**							
Race & gender	869	51.9	426	62.4	21.0	1	<0.001^*^
Black/Latinx	203	12.1	113	16.5	7.8	1	0.005^*^
Female	772	46.1	364	53.3	9.7	1	0.002^*^
Black	77	4.5	53	7.4	8.3	1	0.004^*^
Latinx	130	7.6	66	9.2	1.729	1	0.189
**Interviews**							
Race & gender	272	58.7	104	73.8	9.7	1	0.002^*^
Black/Latinx	53	11.4	21	14.9	0.9	1	0.344
Female	254	54.9	97	68.8	8.1	1	0.004^*^
Black	20	4.3	10	7.0	1.8	1	0.186
Latinx	33	7.0	11	7.7	0.071	1	0.790
**Offers**							
Race & gender	85	63.0	44	88.0	9.7	1	0.002^*^
Black/Latinx	18	13.3	10	20.0	0.8	1	0.372
Female	79	58.5	40	80.0	6.4	1	0.011^*^
Black	5	3.6	4	8.0	1.5	1	0.219
Latinx	13	9.5	6	12.0	2.53	1	0.615

Race & gender refers to total number of participants who identified as either female, Black, or Latinx (or any combination thereof) collapsed into a single overarching diversity category.

* Statistically significant at the α = 0.017 level based on Bonferroni correction for multiple comparisons.

With regard to overall diversity (race and/or gender combined) of candidates, data revealed a significant increase from pre to post workshop in applicants to positions [χ^2^(1) = 21.0, *p* < 0.001], applicants being offered an interview [χ^2^(1) = 9.7, *p* = 0.002], and those eventually offered a position [χ^2^(1) = 9.7, *p* = 0.002]. Follow-up comparison looking at race (Black and/or Latinx) only found a significant increase from pre to post workshop only in applicants to positions, [χ^2^(1) = 7.8, *p* = 0.005], but not interviews, [χ^2^(1) = 0.9, *p* = 0.33], or offers, [χ^2^(1) = 0.8, *p* = 0.37]. Follow-up comparisons of a further breakdown into specifically Black or Latinx categories indicated the observed increase in applicants was a function of an increase in Black applicants and not Latinx applicants. The follow-up comparison looking only at gender (female) revealed significant increases from pre to post workshop in number of applicants [χ^2^(1) = 9.7, *p* = 0.002], interviews extended to applicants [χ^2^(1) = 8.1, *p* = 0.004], and eventual positions offered [χ^2^(1) = 6.4, *p* = 0.011]. Findings indicated improvements were greater for gender than for race.

**Figure 2:**
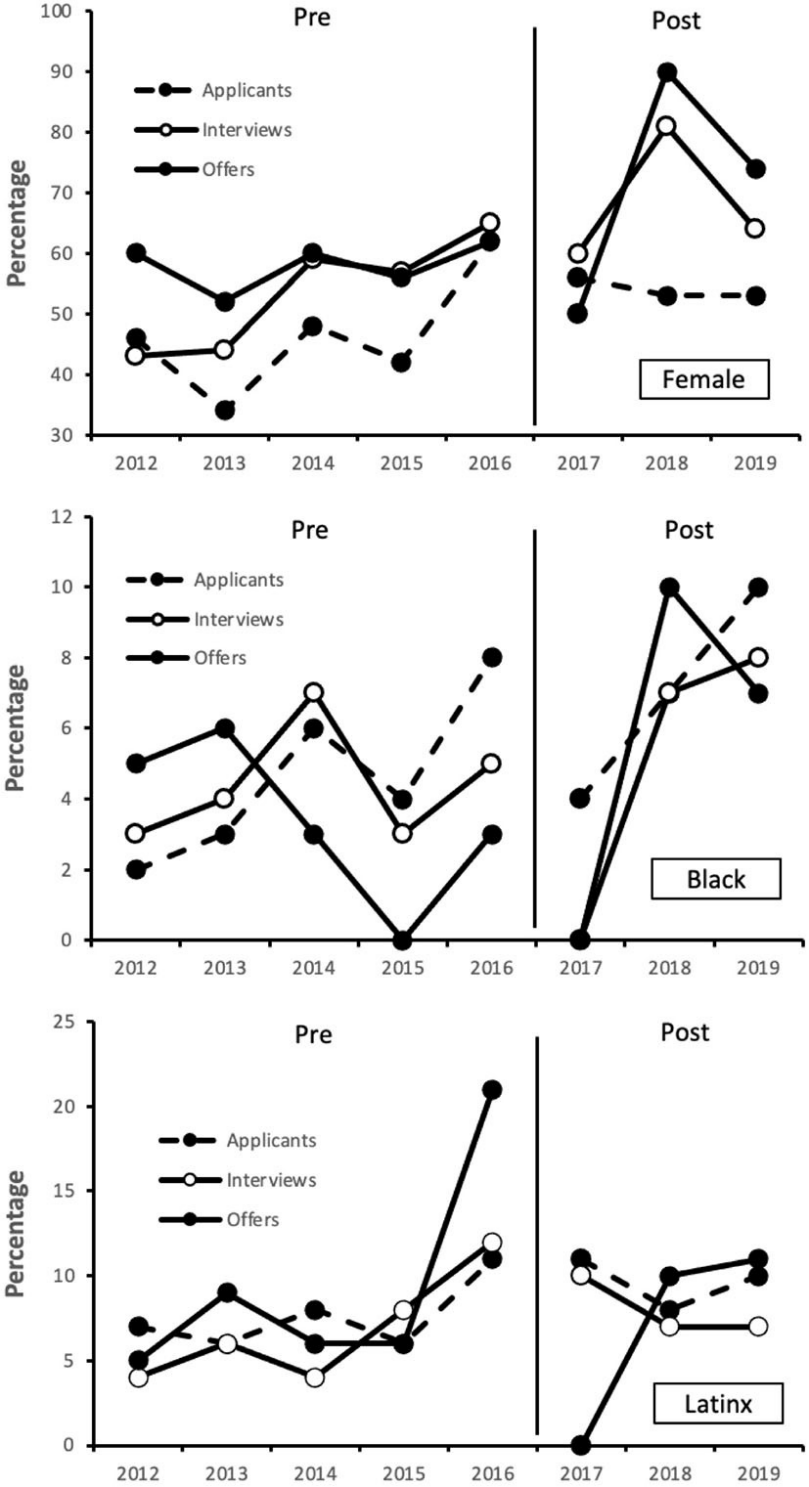
Percentage of diverse applicants, interviewees, and hires per calendar year before and after implementation of search committee workshop.

A depiction of the year-by-year trends for the 5 years prior to and 3 years following implementation of the search committee workshop is presented for each diversity category in Figure 2. Improvements in diversity from pre to post workshop do not seem to be an artifact of natural linear changes over time. It is evident for female individuals that levels of all categories were generally higher following the workshop, especially in the two latter years (2018–2019), despite a slight increase in the final year prior to implemention (2016). A similar increase was observed for both Black and Latinx individuals in the final year before the workshop (2016), followed by a downturn in the first year of the workshop (2017). For Black individuals, a clear increase was observed in the final 2 years of the workshop period (2018–2019), though low sample sizes likely impacted reaching statistical significance (see Table 3). Notably, a similar pattern was not readily apparent for Latinx individuals as it was for female and Black individuals in the post-workshop period.

## Discussion

Many best practices aimed at diversifying the academic medicine workforce are touted in the literature as gold-standard, however, there is a lack of evidence to demonstrate their effectiveness. In the present study, we combined several best practices into one training package and observed a corresponding improvement in diversity of applicants, candidates interviewed, and faculty hired in the period of time after workshop implementation began. The present findings are preliminary in nature because the applied research design employed could not rule out the possible influence of extraneous factors on the significant improvements observed. In addition, our findings also indicate implementation of such a training package is feasible, perceived to be socially valid by participants, and therefore likely worth the investment of time and resources by medical schools.

The training package addresses two of three levels of racism (44), heeding the call by scholars in academic medicine to go beyond implicit bias training and create antiracist practices by addressing systemic racism (45, 46). Systemic racism is addressed by implementing a policy (Faculty Search Policy for Diversity and Excellence) that mandates use of best practices to promote diversity in the search process. Interpersonal racism is addressed *via* mandated implicit bias assessment and training for all members of search committees.

We found an increase in diversity of applicants for gender and race combined as well as when examined separately. However, in terms of diversity of those applicants who were then offered interviews and positions, we observed an increase in overall diversity, when race and gender were combined, as well as with gender only, but not for race when analyzed separately. The significant increase in overall diversity of interviewees and hires was largely driven by the increase in gender as compared with race.

There are several possibilities that could account for the lack of statistically significant increases in racial diversity among those candidates who were selected for both interviews and offers. First, it is possibly due to smaller sample sizes among the Black/Latinx category only, relative to those categories which included gender, that may have precluded the possibility to detect anything other than a very large effect. Additionally, while we are not aware of any empirical data that can be used to support the following notion, it may be possible that increased national awareness of cultural movements such as #MeToo and #TimesUp exerted some level of influence during the timeframe of the present study and may have therefore drawn additional attention to issues of gender equity during that time. It is worth noting these data were collected prior to the more recent focus on systemic racism following high-profile instances of police brutality toward communities of color in 2020. In this vein, another strength of this study is that the training package addressed not only interpersonal bias *via* implicit bias training but also potential institutional and systemic barriers through the practices outlined in the DCL.

Based on our finding that greater improvements were observed among gender relative to race, the workshop has been revised to include a greater emphasis on racial diversity and intersectionality between race and gender. Now that we have demonstrated the feasibility of the workshop, next steps include collaboration and implementation at other medical schools to increase generalizability of results and establish greater experimental control of the workshop intervention. In addition, other data collected during the workshop rollout but not presented here will also be analyzed, in order to determine which best practices contributed most (i.e., component analysis) to the diversity of candidate pools and applicants hired, as well as determine the participatory influence of implicit race and gender bias among search committee members on various outcomes of the search process.

Although the findings are promising, this study was limited by lack of a control group, as the newly adopted Faculty Search Policy to Create Excellence and Diversity mandated participation of all search committees in the newly established process. As such, it is possible the demonstrated changes in diversity of applicants hired could have been due to variables other than the workshop itself, such as the pro-diversity culture at our institution, as reflected in the willingness of our school to adopt the Faculty Search Policy to Create Excellence and Diversity and mandate best practices to increase diversity. Another limitation includes our inability to stratify results by type of position (e.g., clinical vs. research positions or junior faculty vs. leadership positions) due to threats to confidentiality if results were broken down in such a way. Similarly, we were unable to disaggregate results by department due to privacy concerns, so results may have been skewed by departments who were more diverse prior to the search and continued to be after workshop implementation. Lack of ability to further disaggregate data also precluded an evaluation of intersectionality. In addition, generalizability was limited since this study was conducted at only one site. Our low response rate for SV1 and SV2 surveys may have reflected a response bias and specific steps to increase response rates to workshop evaluations should be incorporated in future studies.

Despite the limitations of this naturalistic study, we were able to demonstrate participatory influence of a diversity training package that incorporated best practices to promote diversity at each stage of the search process. Following implementation of the workshops, we noted a significant increase in the diversity of applicants, candidates interviewed, and faculty hired, though we cannot attribute this improvement solely to the workshop at this time. In addition, we demonstrated that the mandatory training package was feasible and well received by participants. Based on these findings, other institutions are informed that requiring search committee members to participate in diversity training and mandating best practices is well worth their investment of time, effort, and resources.

## Data availability statement

The raw data supporting the conclusions of this article will be made available by the authors, without undue reservation.

## Ethics statement

The studies involving human participants were reviewed and approved by the University of Nevada, Reno Institutional Review Board reviewed this protocol and deemed the study to be Exempt from IRB Review on 6 March 2017, Reference # 980480-1. The patients/participants provided their written informed consent to participate in this study.

## Author contributions

NJ wrote the introduction and discussion, developed and carried out the trainings, and oversaw data collection. JE-L helped to develop and carry out the trainings, directly collected data, and oversaw data analysis along with our office of continuous institutional assessment. GS wrote the methods and results section, helped with data analysis, contributed significantly to writing of the introduction, and methods as well as edits to the overall manuscript. MP provided substantive edits to the overall manuscript. RH oversaw the project, had input on methods, and helped to edit the overall manuscript. All authors contributed to the article and approved the submitted version.

## Conflict of interest

The authors declare that the research was conducted in the absence of any commercial or financial relationships that could be construed as a potential conflict of interest.

## Publisher's note

All claims expressed in this article are solely those of the authors and do not necessarily represent those of their affiliated organizations, or those of the publisher, the editors and the reviewers. Any product that may be evaluated in this article, or claim that may be made by its manufacturer, is not guaranteed or endorsed by the publisher.
